# Use of name recognition software, census data and multiple imputation to predict missing data on ethnicity: application to cancer registry records

**DOI:** 10.1186/1472-6947-12-3

**Published:** 2012-01-23

**Authors:** Ronan Ryan, Sally Vernon, Gill Lawrence, Sue Wilson

**Affiliations:** 1 Public Health, Epidemiology and Biostatistics, University of Birmingham, Birmingham B15 2TT UK; 2 Eastern Cancer Registration and Information Centre, Public Health Building, Unit C - Magog Court, Shelford Bottom, Hinton Way, Cambridge, CB22 3AD UK; 3 West Midlands Cancer Intelligence Unit, Public Health Building, University of Birmingham, Birmingham B15 2TT UK; 4 Primary Care Clinical Sciences, University of Birmingham, Birmingham B15 2TT UK

## Abstract

**Background:**

Information on ethnicity is commonly used by health services and researchers to plan services, ensure equality of access, and for epidemiological studies. In common with other important demographic and clinical data it is often incompletely recorded. This paper presents a method for imputing missing data on the ethnicity of cancer patients, developed for a regional cancer registry in the UK.

**Methods:**

Routine records from cancer screening services, name recognition software (Nam Pehchan and Onomap), 2001 national Census data, and multiple imputation were used to predict the ethnicity of the 23% of cases that were still missing following linkage with self-reported ethnicity from inpatient hospital records.

**Results:**

The name recognition software were good predictors of ethnicity for South Asian cancer cases when compared with data on ethnicity derived from hospital inpatient records, especially when combined (sensitivity 90.5%; specificity 99.9%; PPV 93.3%). Onomap was a poor predictor of ethnicity for other minority ethnic groups (sensitivity 4.4% for Black cases and 0.0% for Chinese/Other ethnic groups). Area-based data derived from the national Census was also a poor predictor non-White ethnicity (sensitivity: South Asian 7.4%; Black 2.3%; Chinese/Other 0.0%; Mixed 0.0%).

**Conclusions:**

Currently, neither method for assigning individuals to an ethnic group (name recognition and ethnic distribution of area of residence) performs well across all ethnic groups. We recommend further development of name recognition applications and the identification of additional methods for predicting ethnicity to improve their precision and accuracy for comparisons of health outcomes. However, real improvements can only come from better recording of ethnicity by health services.

## Background

This paper presents a method for imputing missing data on the ethnicity of cancer patients, developed for a regional cancer registry in the UK. It implements existing approaches in a novel situation and evaluates their utility. It combines four differing approaches to dealing with missing data of this type: the use of an additional source of self-reported ethnicity to replace the missing data; the use of name recognition software to predict the ethnicity of individuals; the use of Census data based on area of residence to predict the ethnicity of individuals; and finally, the use of multiple imputation (MI) to make an allowance for the use of these predictors in subsequent statistical analyses. The method has applications beyond cancer registries, and the results presented below are relevant to all organisations and researchers that have incomplete information on the ethnic group of individuals who have access to additional data that may help predict ethnicity when missing.

The West Midlands Cancer Intelligence Unit (WMCIU) is a regional cancer registry covering a population of approximately 5.3 million. The registry collects information about new cases of cancer and produces statistics about incidence, prevalence, survival and mortality: the availability of information on the sociodemographic characteristics, including ethnicity, of cancer cases is important for service planning, ensuring equal access to these services, and for epidemiological studies. The main source of information on the ethnicity of cancer cases for the WMCIU is linkage to the national Hospital Episode Statistics database (HES), which routinely collects the self-reported ethnicity of hospital patients [1]. However, HES currently only provides information on patients admitted to hospital, and does not include patients who were assessed or treated in secondary care, but not admitted, nor does it include information on patients seen privately outside the National Health Service (NHS). Nationally, only 80% of cancer cases have ethnicity data available from HES [2]. This reliance on linkage with hospital admissions has implications for cancer incidence, prevalence and survival. The use of complete case analyses in this situation has the potential to cause bias: for example, cases of prostate cancers who are not treated ('watchful waiting') may have longer survival than those whose cancer is more advanced and are therefore admitted to hospital. The exclusion of the former from survival analyses will tend to underestimate survival overall, and, if ethnicity is associated with treatment type, may obscure any differences in survival between ethnic groups. Similarly, cancer cases who are known to the WMCIU through death certification only (DCO) (the only information they hold about the case is based on a death certificate) have less complete linkage with HES. Any comparison of cancer incidence by ethnic group restricted to complete cases has the potential to obscure any differences between ethnic groups if people from ethnic minorities are over represented among the DCO cases.

There are alternative approaches to dealing with missing information that allow all cases to be retained for analysis. The simplest of these is to link with additional external information sources that record ethnicity, such as the National Breast Cancer Screening Service (NBSS), but the population coverage, completeness and accuracy of these data may also be limited. A second commonly used approach is to use lists of names that are associated with particular ethnic groups. Name recognition software packages such as Nam Pehchan and SANGRA have been used in a variety of settings to identify people with South Asian heritage [3-6]. More recently, another package called Onomap has become available: this attempts to identify people from many ethnic groups [7,8]. Each of these packages relies on individuals having a name that is strongly associated with a particular ethnic group and their sensitivity and specificity is known to vary from setting to setting [9-11]. None of them can identify people who would describe themselves as having a mixed ethnicity, individuals whose surnames are not specific to ethnic groups, or the original ethnic group of individuals who adopt their partner's surname where the partner is a member of another ethnic group. Another approach is the use of census information on the ethnic distribution of the area in which the case lived. Examples of this are a study on the uptake of breast cancer screening services in London, and the development of a risk calculator for coronary heart disease based on data from a large set of general practices [12,13]. This approach has the advantage of being easy to implement if the postcode of the individual is available, but relies on the assumption that the ethnic distribution of a Census area (approximately 1,500 people) is an accurate predictor of the ethnicity of individuals.

The use of MI, such as that implemented by Royston and colleagues in the statistical software package Stata, has the potential to create a complete dataset that combines the predictions generated by the above methods, and makes an allowance for the imprecision of these predictions that is carried through to the final statistical analyses [14]. It requires the user to have an accurate understanding of the reasons why the data are missing (the missing data mechanism), good predictors of the value of the missing data, and assumes that the data are otherwise missing at random (MAR). It is, therefore, a combined approach which aims to maximise accuracy where missing values are predicted and to adjust the precision of any estimates derived from these predictions (e.g. by widening the confidence intervals on estimates of cancer incidence).

We will present information on the sensitivity and specificity of each of these methods, and describe how these multiple sources were combined in a dataset that can be used for the estimation of cancer incidence and survival by ethnic group.

## Methods

### Population

111 694 cancer cases normally resident in the West Midlands region of the UK and diagnosed between 1 January 2001 and 31 December 2007 were included in the cohort for this project. Cases were limited to the five most common cancer sites (breast, upper GI, lower GI, prostate, and lung) as there would be too few cases from the non-White ethnic group to allow reliable comparisons of incidence and survival for the less common cancers.

### Ethnic groups

The five ethnic groupings used were: White, South Asian, Black, Chinese/Other, and Mixed. These broad groupings coincide with those used in official national statistics [15].

### Availability of existing data on ethnicity

Data on ethnicity were available for 85 506/111 694 (77%) of cancer cases from HES, although some individuals had more than one ethnic group recorded. Where this occurred (1506/85 506 (1.8%)), we used the most commonly recorded ethnic group, in line with the method recommended by Downing and colleagues [16]. This approach is believed to be appropriate as it uses most information. We set ethnic group to missing for cases with more than one ethnic group recorded, but without a 'most common' ethnic group (154/85 506 (0.18%) of the complete cases), again in line with the method used by Downing. We then tabulated the characteristics of the cohort, and used a univariate chi-square analysis to identify demographic and clinical factors associated with missing ethnicity (the missing data mechanism) [17,18].

### Additional source of ethnicity data

The 28 795 breast cancer cases from the cohort were linked to data held by the eight breast cancer screening services (NBSS) in the region using their NHS number. We assessed the value of this additional information by comparing sensitivity, specificity and positive predictive value of the ethnicity recorded in the NBSS using the HES dataset as the gold standard, where both were available.

### Prediction of ethnicity using name recognition software

Two name recognition applications were available for use in this project: Nam Pehchan and Onomap. Nam Pehchan was used to identify people with South Asian names, and Onomap was used to identify people with names associated with White, South Asian, Black and Chinese/Other ethnic groups. As early use of Nam Pehchan with this cohort showed that it included forenames which were common among other ethnic groups (e.g. 'Mona'), we decided to run it on forenames and surnames separately, rather than the default approach which was to run it on all forenames and surnames combined. This allowed matched surnames to carry a greater weight than matched forenames in the MI process.

Since Onomap only makes use of a single forename, only the first forename was used when the application was run. However, as cases could have more than one surname associated with their WMCIU record, the application was run with each combination of forename and surname for each case. If Onomap assigned a case to more than one broad ethnic group, their multiple results were replaced by a single result according to the following order of preference: Chinese/Other, Black, South Asian, White. This ordering corresponds with the relative size of each group within the regional population, with preference given to the less common ethnic groups. The sensitivity, specificity, and positive predictive value (PPV) of the two applications was compared with the ethnic group recorded in HES in order to assess the ability of each to identify the ethnicity of cancer cases.

### Prediction of ethnicity using area-based Census data

Cases were assigned to the Census area (lower layer super output area (LSOA): average size 1500 persons) associated with their postcode or residence, and linked to a dataset with the ethnic distribution of each LSOA in the 2001 national Census.

### Full multiple imputation model

The last stage in processing, the imputation of the missing ethnicity using the existing variables shown to be associated with missing ethnicity (Table 1) and external information derived from the above sources was carried out in Stata using the MI package ICE [14]. The linked NBSS data was used directly to replace the ethnic group of cases not already known from linkage with HES, rather than as a separate predictor in the imputation model. Where the case's surname at birth was available (from their death certificate) the Onomap and Nam Pehchan results for that name were used in place of the results for all known names in the imputation model, as name at birth may be a more accurate reflection of ethnicity for individuals who have changed their name following marriage. In addition, as we intended to use the data for cancer-specific and all-cause mortality survival analyses, we included these survival outcomes as covariates in the imputation model, along with the time to each outcome (the Nelson-Aalen estimate of the cumulative hazard function) [14]. The number of imputed datasets was chosen conservatively: one imputed dataset per 1% of cases with any missing data. Missing ethnicity was imputed using multinomial logistic regression within the ICE package, and the distribution of imputed values was tabulated for comparison with the observed (complete case) data. The sensitivity, specificity, and positive predictive value (PPV) of the full multinomial logistic model was compared with the ethnic group recorded in HES in order to assess its ability to identify the ethnicity of cancer cases. The model was developed on a randomly selected 50% sample of the 85352 cases whose ethnicity was recorded in the HES dataset. The remaining 50% of cases were used to validate the model and derive the above estimates. The predictors used in the model were: ethnicity derived from name recognition software; Census estimates of ethnic distribution of population; number of hospital admissions; year of diagnosis; patient seen outside the NHS (yes/no); screen-detected cancer (yes/no); death certificate only cancer registration (yes/no); cancer treatment type (surgery/radiotherapy/chemotherapy); deprivation score; gender; age at diagnosis; cancer site; and death during follow-up period (all-cause and due to primary cancer separately) and time to death/censoring (Nelson-Aalen cumulative hazard).

**Table 1:** Characteristics of the Project Cohort

		Cases	Cases with missing ethnicity following linkage with HES records (% of cases)	Chi2 statistic (*P*-value)
Site	Lower GI	24 446	4112(17)	
	Breast	28 795	6029(21)	
	Lung	24 060	5660(24)	
	Prostate	23 716	8814(37)	
	Upper GI	10 677	1727(16)	3500(p < 0.001)
				
Year of diagnosis	2001	15 102	4118(27)	
	2002	15 523	3840(25)	
	2003	15 731	3772(24)	
	2004	16 162	3681(23)	
	2005	16 317	3632(22)	
	2006	16 458	3470(21)	
	2007	16 401	3829(23)	206(p < 0.001)
				
Deprivation	1 (most deprived)	26 738	5149(19)	
(Income Domain of	2	22 104	4856(22)	
Index of Multiple	3	22 759	5425(24)	
Deprivation 2007)	4	22 465	5942(26)	
	5 (least deprived)	17 628	4970(28)	6300(p < 0.001)
				
Age	< 40	1771	251(14)	
	40-49	5622	892(16)	
	50-59	15 338	2933(19)	
	60-69	27 759	5924(21)	
	70-79	35 258	8538(24)	
	80+	25 946	7804(30)	1100(p < 0.001)
				
Sex	Male	59 592	15 454(26)	
	Female	52 102	10 888(21)	391(p < 0.001)
				
Death Certificate	No	106 217	23 577(22)	
Only registration	Yes	5477	2765(50)	2300(p < 0.001)
				
Ever seen privately	No	106 566	23 113(22)	
(cancer was diagnosed or treated outside the free National Health Service at least on one occasion)	Yes	5128	3229(63)	4600(p < 0.001)
				
Surgery	No	58 875	18 344(31)	
	Yes	52 819	7998(15)	4000(p < 0.001)
				
Radiotherapy	No	73 520	19 009(26)	
	Yes	38 174	7333(19)	616(p < 0.001)
				
Chemotherapy	No	93 778	24 650(26)	
	Yes	17 916	1692(9)	2400(p < 0.001)
				
Screen detected	No	22 900	5012(22)	
breast cancer*	Yes	5895	1017(17)	61(p < 0.001)
				
HES-linked	No	19 694	19 694(100)	
	Yes	92 000	6648(7)	
				
Number of admissions	0	19 694	19 694(100)	
(includes non-cancer admissions)	1	8012	2071(26)	
	2	10 261	1414(14)	
	3	10 523	985(9)	
	4	9332	562(6)	
	5+	53 872	1616(3)	6300(p < 0.001)**

* Comparison of breast cancer cases who were and were not detected by population screening.

** Chi-square test excludes cases with no admissions as, by definition, none have ethnicity recorded.

### Research governance

The project did not require separate ethical approval as it was commissioned by, and carried out in collaboration with, the regional cancer registry. Cancer registries have legal support to collect data relating to cancer under Section 251 of the NHS Act 2006 (and formerly under Section 60 of the Health and Social Care Act 2001).

[http://www.ukcancassoc.ismysite.co.uk/content/legal-background#S251].

## Results

The completeness of information on the ethnicity of cancer cases following linkage with HES varied significantly (*P *< 0.001 in each case) by the demographic and clinical factors listed in Table 1.

The value of linkage with breast cancer screening services (NBSS) information on ethnicity is shown in Table 2 and Table 3. Table 2 describes the sensitivity, specificity and PPV of ethnicity derived from the NBSS compared with that recorded in HES (i.e. using HES as a gold standard), for 5243 breast cancer cases with ethnic group recorded in both HES and NBSS datasets. Sensitivity was high (> 90%) for White and South Asian cases, and moderately high (61.4%) for Black cases, suggesting that the NBSS could be used to determine the ethnicity of cancer registry cases where this was not recorded in HES. No cases recorded as Chinese/Other or Mixed ethnicity in HES were assigned the same ethnic group in the NBSS dataset. However, the value of the NBSS records for these two ethnic groups cannot be precisely determined because of the small numbers involved (14 and 10 cases, respectively). Table 3 shows the effect of using the NBSS data to resolve the ethnicity of registry cases that were not recorded in HES. A total of 1082/26 342 (4.1%) breast cancer cases whose ethnicity was not known in HES had an ethnic group recorded in the NBSS. Overall it decreased the proportion of cancer cases with unknown ethnicity from 23.6% to 22.6%.

**Table 2:** Sensitivity, Specificity and Positive Predictive Value of NBSS-derived Ethnicity for Breast Cancer Cases

Ethnic group recorded in HES	Number of cases recorded in HES	NBSS		
		Sensitivity	Specificity	Positive predictive value
White	5093	99.7%	77.3%	99.3%
South Asian	82	90.0%	99.8%	87.1%
Black	44	61.4%	99.9%	79.4%
Chinese/Other	14	0.0%	100.0%	
Mixed	10	0.0%	100.0%	

Includes 5243 cases where ethnic group was recorded in both HES and NBSS datasets. Individual logistic models (positive outcome threshold: p > = 0.5).

**Table 3:** Ethnicity of Cases Following Linkage with HES and NBSS Datasets

Ethnic group	Cases with ethnic group recorded in HES (%)		Cases with unknown ethnicity resolved by NBSS linkage	Ethnic breakdown of cohort following use of HES and NBSS (%)	
White	81 934	(73.4)	1053	82 987	(74.3)
South Asian	1545	(1.4)	13	1558	(1.4)
Black	1429	(1.3)	11	1440	(1.3)
Chinese/Other	303	(0.3)	3	306	(0.3)
Mixed	141	(0.1)	2	143	(0.1)
					
Not known	26 342	(23.6)		26 331	(22.6)
					
Total	111 694		1082		

The sensitivity, specificity and PPV of Onomap and Nam Pehchan for each ethnic group is shown in Table 4. The sensitivity of Onomap is high for White and South Asian ethnic groups (99.8% and 82.1%), but low for Black and Chinese/Other groups (4.4% and 0.0%). The sensitivity of Nam Pehchan was lower that of Onomap for South Asian cases (71.1% and 82.1%), but when both were combined, sensitivity was higher than each individual application (90.5%). A total of 14 615 cases had their name at birth recorded on their death certificate.

**Table 4:** Sensitivity, Specificity and Positive Predictive Value of Name Recognition Software

Name recognition software	Ethnic group	Sensitivity	Specificity	Positive predictive value
Onomap	White	99.8%	51.5%	98.0%
	South Asian	82.1%	99.9%	92.9%
	Black	4.4%	99.9%	70.8%
	Chinese/Other	0.0%	100.0%	
				
Nam Pehchan*	South Asian	71.1%	99.9%	94.5%
				
Onomap and Nam Pehchan combined	South Asian	90.5%	99.9%	93.3%

Includes 85 352 cases where a single ethnic group was recorded in the HES dataset. Individual logistic models (positive outcome threshold: p > = 0.5).

* Matched on forename and surname separately.

Table 5 shows the sensitivity, specificity and PPV of 2001 national Census data on ethnicity as a predictor of the ethnic group of individual cases. The sensitivity of Census data for the White ethnic group is high (99.3%), but very low for all other ethnic groups (less than 7.4% for South Asian cases, 2.3% for Black cases, and 0% for the remaining two groups).

**Table 5:** Sensitivity, Specificity and Positive Predictive Value of Census Data on Ethnicity

Ethnic group	Sensitivity	Specificity	Positive predictive value
White	99.3%	21.4%	96.8%
South Asian	7.4%	99.8%	44.9%
Black	2.3%	99.9%	34.4%
Chinese/Other	0.0%	100.0%	
Mixed	0.0%	100.0%	

Includes 85352 cases where a single ethnic group was recorded in the HES dataset. Census data used to predict ethnic group were: percentage of local population in South Asian, Black, Chinese/Other and Mixed ethnic group at last national census. Individual logistic models (positive outcome threshold: p > = 0.5).

The ethnicity of cases that were missing following linkage with the HES and NBSS datasets was imputed in Stata using ICE with an imputation model that included the variables significantly associated with missingness (Table 1), the predicted ethnicity of each case made using Onomap and Nam Pehchan, and the ethnic breakdown of the area of residence of the case. The number of imputed datasets generated for the full run was set to 23 as ethnicity was missing for 22.6% of the cases (Table 3).

Table 6 shows the sensitivity, specificity and PPV of the full multinomial logistic regression model used to impute missing ethnicity. The sensitivity and specificity of the full model was comparable to that from the name recognition software alone for the White group (99.3%/56.0% vs. 99.8%/51.5%, respectively). The sensitivity of the full model was slightly higher for cases from the South Asian group than name recognition software alone (94.7% vs. 90.5%, respectively), and substantially higher for Black and Chinese/Other ethnic groups (20.4% vs. 2.3% and 21% vs. 0%, respectively). The sensitivity of the full model for the Mixed ethnic group remained at 0%.

**Table 6:** Sensitivity, Specificity and Positive Predictive Value of Full Model

Ethnic group	Sensitivity	Specificity	Positive predictive value
White	99.7%	56.0%	98.2%
South Asian	94.7%	99.8%	90.4%
Black	20.4%	99.8%	63.6%
Chinese/Other	21.0%	99.9%	57.6%
Mixed	0%	100%	

A multinomial logistic regression model was used to predict ethnic group. The model was developed on a randomly selected 50% sample of the 85352 cases whose ethnicity was recorded in the HES dataset. The remaiming 50% of cases were used to validate the model and derive the above estimates. The predictors used in the model were: ethnicity derived from name recognition software; Census estimates of ethnic distribution of population; number of hospital admissions; year of diagnosis; patient seen outside the NHS (yes/no); screen-detected cancer (yes/no); death certificate only cancer registration (yes/no); cancer treatment type (surgery/radiotherapy/chemotherapy); deprivation score; gender; age at diagnosis; cancer site; and death during follow-up period (all-cause and due to primary cancer separately) and time to death/censoring (Nelson-Aalen cumulative hazard).

Table 7 compares the proportion of cases in each ethnic group for complete and imputed cases (all 23 imputations combined). The proportion of cases in the White, South Asian and Black groups was slightly lower among the imputed cases than the complete cases (95.8% vs. 96%, 1.7% vs. 1.8%, and 1.6% vs. 1.7%, respectively). For the remaining ethnic groups, the proportion of cases each group was approximately 1.5 times higher among the imputed cases (0.6% vs. 0.4%, and 0.3% vs. 0.2% for Chinese/Other and Mixed ethnic groups, respectively.)

**Table 7:** Comparison of Distribution of Ethnic Groups: Observed and Imputed

Ethnic group	Observed %	Imputed* %	Total* %
White	96.0	95.8	96.0
South Asian	1.8	1.7	1.8
Black	1.7	1.6	1.7
Chinese/Other	0.4	0.6	0.4
Mixed	0.2	0.3	0.2
			
Total	100	100	100

* Using all 23 imputations combined.

## Discussion

The main aim of this project was to create a method to impute the ethnic group of cancer cases who were notified to the regional cancer registry, but whose ethnic group was not available from their main source, linkage with the national database on hospital admissions (HES). We made use of precise external information on the ethnicity of cases where possible, through linkage with a further dataset (the NBSS), two name recognition applications, and area-based information on the ethnic make-up of the resident population. We then assessed the value of each of these additional sources by comparing them with the ethnic group of cases whose ethnicity was known from HES. In the final stage of the method, we created a dataset which can be used to estimate ethnic group specific cancer incidence and survival: this involved the use of a MI procedure (ICE).

The main benefit of using additional linked datasets, like the NBSS, is that it makes use of precise information recorded about the individuals of interest. The main limitation for this project is that the NBSS dataset only contains breast cancer cases who attended the screening programme and who had their ethnic group recorded at that time: this resolved the ethnic group of just 1% of the cancer cases whose ethnicity was not already known from linkage with HES. We decided to use NBSS recorded ethnicity as a direct substitute for missing ethnicity rather than including it as a predictor of ethnicity in the MI process: it did refer directly to the person of interest. Similar datasets were not available for the other cancer sites of interest.

The performance of Nam Pehchan is widely known but, as far as we are aware, this is the first peer reviewed report on the Onomap application. The higher sensitivity and specificity that was achieved by using both applications together suggests that the best name-based predictions of ethnicity can be achieved by the use of multiple applications. It is, however, unlikely that name recognition software will ever precisely predict membership of some ethnic groups: although many people from South Asian, Chinese and some other ethnic groups may have distinctive names, many individuals from White, Black and Mixed ethnic groups do not. This suggests that we will always have to make some allowance for their imprecision, and include additional predictors of ethnicity.

Area-based information from the national Census is a popular predictor of ethnicity and easy to implement, but this project demonstrates that is not precise enough to be used alone. Although sensitivity for the White ethnic group may be high (> 99%) specificity is very low (21%), showing that it misclassifies approximately 4 out of every 5 people from non-White backgrounds as White. Similarly, sensitivity is poor for the most common non-White ethnic groups in the region: the use of area-based Census data only appears to correctly identify approximately 7% of South Asian and 2% of Black cases. Setting alternative cutoff values for the model predictions from the default value of 0.5 did not improve the predictive performance of the Census data to any great extent (results not shown).

The full model used to predict ethnicity within the MI procedure did appear to be superior to naming software and Census data alone. The model appeared to perform best for the South Asian ethnic group, and did identify membership of the White, Black and Chinese/Other ethnic groups with greater sensitivity than any of its individual constituent predictors. However, the sensitivity of the full model in absolute terms for Black, Chinese/Other and Mixed groups is low. It is, therefore, uncertain that the existing predictors can be improved, or that new predictors could be added, which would increase the sensitivity of future models to levels similar to that seen for the South Asian group.

The greatest difference between the observed and imputed data in the final model was for two of the three minority groups whose ethnicity is poorly predicted by name recognition software (Chinese/Other and Mixed). This may indicate that the imputation process does not perform well in these cases. New MI models may benefit from including other predictors of ethnicity which help identify membership of these groups more precisely, using age-specific data on the ethnic composition of small geographic areas (LSOAs) if this is published following the next national Census or country of birth, for example.

## Conclusions

In summary, we have developed a method and dataset that will allow comparison of cancer incidence and survival between ethnic groups. However, the sensitivity of the Onomap name recognition application appears to be low for people from non-White and non-South Asian ethnic groups, suggesting that it is of limited use for studies that wish to classify individuals by ethnic group. Similarly, area-based information from the national Census, a common approach where individual names are not available but area of residence is, appears to be imprecise, particularly for the less common ethnic groups. Currently, neither method for assigning individuals to an ethnic group performs well across all ethnic groups. We recommend further development of name recognition applications and the identification of additional methods for predicting ethnicity to improve their precision and accuracy for comparisons of health outcomes. Nevertheless, neither imputation nor name recognition software will be completely accurate: reliable statistics relating to the incidence, prevalence and survival of persons with cancer by ethnic group require more complete recording of these data [19].

## Competing interests

The authors declare that they have no competing interests.

## Authors' contributions

All authors contributed to the design of the project. RR led the development of the method for imputing missing data, performed the analysis, and wrote the first draft of the manuscript. All authors contributed to later drafts and approved the final manuscript.

## Pre-publication history

The pre-publication history for this paper can be accessed here:

http://www.biomedcentral.com/1472-6947/12/3/prepub
